# Local botanical knowledge of native food plants in the semiarid region of Brazil

**DOI:** 10.1186/s13002-018-0249-0

**Published:** 2018-07-20

**Authors:** Ernane N. Nunes, Natan M. Guerra, Edna Arévalo-Marín, Carlos Antônio B. Alves, Viviany T. do Nascimento, Denise D. da Cruz, Ana H. Ladio, Silvanda de M. Silva, Rodrigo S. de Oliveira, Reinaldo F. P. de Lucena

**Affiliations:** 1 Laboratório de Etnobiologia e Ciências Ambientais, Departamento de Sistemática e Ecologia, da Universidade Federal da Paraíba, Campus I, João Pessoa, Paraíba 58.051-900 Brazil; 2 Laboratório de Química de Biomassa, Departamento de Engenharia Química, da Universidade Federal de Campina Grande, Campus Campina Grande, Campina Grande, Paraíba 58.429-900 Brazil; 3 Programa de Pós-Graduação em Agronomia, da Universidade Federal da Paraíba, Campus II, Areia, Paraíba 58.397-000 Brazil; 4 Subdireção Científica, Jardim Botânico José Celestino Mutis, Avenida Calle 63 No. 68-95, Bogotá D.C., Bogotá Colombia; 5 Laboratório de Ecologia Terrestre, Departamento de Sistemática e Ecologia, da Universidade Federal da Paraíba, Campus João Pessoa, João Pessoa, Paraíba 58059-900 Brazil; 6 Universidade Estadual da Paraíba, Campus Guarabira, Guarabira, Rodovia PB-75, km 01, Bairro, Areia Branca, Guarabira, Paraíba 58.200-000 Brazil; 7 Departamento de Ciências Humanas, Universidade do Estado da Bahia, Campus IX, Rodovia BR 242, Loteamento Flamengo, Barreiras, Bahia 47802-470 Brazil; 8 Laboratorio Ecotono. INIBIOMA, Universidad Nacional del Comahue, Quintral S/N Barrio Jardín Botánico (8400), San Carlos de Bariloche, Río Negro Argentina

**Keywords:** Brazilian semiarid, Native foods, Rural communities, Traditional knowledge, Botanic knowledge

## Abstract

**Background:**

This study aimed to investigate the local botanical knowledge of native food plants in three rural communities, located in the semiarid region of Paraíba State, Brazil, verifying possibilities of differences of knowledge among communities and between men and women.

**Methods:**

Semi-structured interviews about native plant knowledge and use were conducted with all householders in each community, totaling 117 informants. The species similarity among the communities of Pau D’Arco, Várzea Alegre, and Barroquinha was compared with Jaccard index, and the use value index (UV_general_, UV_current_, UV_potential_) was used to determine the most important species. The Kruskal-Wallis test was used to compare the use values among communities and genders. The consensus factor among the informants was calculated according to the uses cited, and the Wilcoxon test was used to compare the use values between men and women.

**Results:**

We recorded 9 species belonging to 8 genera and 8 families in Várzea Alegre; 10 species, 9 genera, and 9 families in Barroquinha; and 7 species, 7 genera and 7 families in Pau D’Arco. *Spondias tuberosa* Arruda (Anacardiaceae) in Várzea Alegre, *Spondias* sp. (Anacardiaceae) in Barroquinha, and *Ximenia americana* L. (Olacaceae) in Pau D’Arco were the most prominent species. Preparation methods are slightly different in the three communities, and there is low similarity about species use among the communities. Regarding gender, the analysis of use value among the communities evidenced significant differences only for UV_general_ among women, specifically between Barroquinha and Pau D’Arco. For men and women within each community, there is a difference only for UV_potential_ in Barroquinha.

**Conclusion:**

This study showed that the residents of the three rural communities have limited knowledge of native food plants found in their communities, but they know where to find them, which parts they may use and how to consume them. The fact is that men know plants that are more distant from the residences and women know those that are next to them.

## Background

In many ancient civilizations, food strongly influenced the culture. It was present in mystical religious rituals, and its importance was evidenced in cave paintings, decorations of archaeobotanical artifacts, pre-Columbian codices, and in many forms of artistic and symbolic representations, demonstrating the abundance and variability of food base at different times [1, 2]. The colonization process also influenced food species consumption. Food plants currently found in the Americas are from Eurasia, such as pea (*Pisum sativum* L.), fava bean (*Vicia faba* L.) [3], and rice (*Oryza sativa* L.) [4], and they were brought to the American continent in the early stages of the Spanish colonization, complementing the food diversity available to people.

Until today, all these native or non-native species are part of the food diversity, and their use is influenced by many factors, including sociocultural, ecological, environmental and scientific factors; however, the cultural inertia associated with the traditions is a determining factor for the consumption of these plants in various communities [5, 6]. In each one of these societies (urban, rural, indigenous), native food plants are also important from a socio-economic point of view, because these resources are an additional nutritional value and are linked to the adaptive processes that led the inhabitants to eat them, and a change in these eating habits may cause socioeconomic stress, mainly driven by a loss of cultural identity [7, 8].

Native species have been intensively studied due to their wide range of phytochemicals with functional properties [9, 10], including phenolic compounds, phytosterols, and terpenes, which are recognized for their benefits to human health, especially for their effects on the metabolic syndrome [11]. As an example, we can mention the nutritional health benefits, promoted by fruit from species belonging to the family Cactaceae, associated with the antioxidant properties related to the presence of ascorbic acid in the fruit, which contains a mixture of yellow betaxanthin, and red betacyanin pigments well known for their anti-cancer properties [12].

Other examples of uses of native food plants are found in Northeast Brazil, where the most important plant families related to human diet, such as Anacardiaceae, Cactaceae, and Euphorbiaceae (species generally encountered and mentioned in dry regions), have been documented in several ethnobotanical studies [13–18]. However, there are few studies documenting food resources used by dryland communities in different regions of the world, which leads to a gap in the knowledge of the used plants, since these communities live with few or no resources to develop agriculture, which creates a food deficit, but even so, the native vegetation and its nutritional possibilities are usually well-known by these communities [5, 19].

On the other hand, over the years, agriculture and yours transitions, how machines, chemical defensive and novel varieties, have provided for the human population a large amount of domesticated and semi-domesticated food species [20], which combine to the wild species consist of a valuable range of possibilities.

According to data from the United Nations Organization for Food and Agriculture, hunger affected almost a billion people all over the world in 2013 [21]. The increase and diversification of agricultural production are one of the solutions commonly pointed to minimize this problem, especially with the discovery and study of native plants, the political will, and humanitarian aid. For each of these possible solutions, there are factors that hinder their implementation and prevent improvements for the poorest populations [13].

However, several plants that once considered part of the food base fell into disuse because of the competition with exotic and processed products, or due to other transculturation processes that have occurred from agricultural revolutions, aforementioned [22]. As a result, the old food management practices, consumption, and preparation methods have been lost over the years [23]. Land use, its availability, and access to wild resources have also changed. Additionally, several investigations have shown that gender is an important factor in determining differences related to the use of wild food plants; according to these studies, women who are in charge of the family diet have a significant role [7, 24, 25].

An analysis of the literature on Latin America shows that the gender division of labor, gendered access to a garden or natural resources, and gendered control over subsistence, and cash crops and income derived from them are crucial factors. In addition, social status related to the gathering practices, gendered knowledge distribution, and transmission are also important parameters [26–30].

Knowledge of food plants is part of the ethnobotanical knowledge, which consists of a kind of knowledge that directly depends on the relationship between societies and vegetation [31]. Some studies have shown that such knowledge is not uniformly present in society because different species have different uses according to the role they play in people’s lives and the practical knowledge that can still be transmitted (or not) by the local culture [27, 32].

In this sense, people can evidence knowledge of plants, but many of them for various reasons do not employ their knowledge of plants [24, 33]. Researchers have interpreted this disconnection between knowledge and practice as the element that causes vulnerability, given that the societies could be losing *biocultural* diversity, disabling self-sufficient ways of making use of the surrounding environment [5, 34, 35].

Based on this context, we investigated three rural communities in the municipalities of São Mamede, Lagoa, and Itaporanga, in Paraíba State (Northeast Brazil), recording and analyzing the local botanical knowledge and the use of native food plants in the semiarid region. The paper focused only in knowledge about plants used as food resource and its preparation forms. In additional, hypothesis was that women and men have a different knowledge.

## Methods

### Study area

**Fig. 1:**
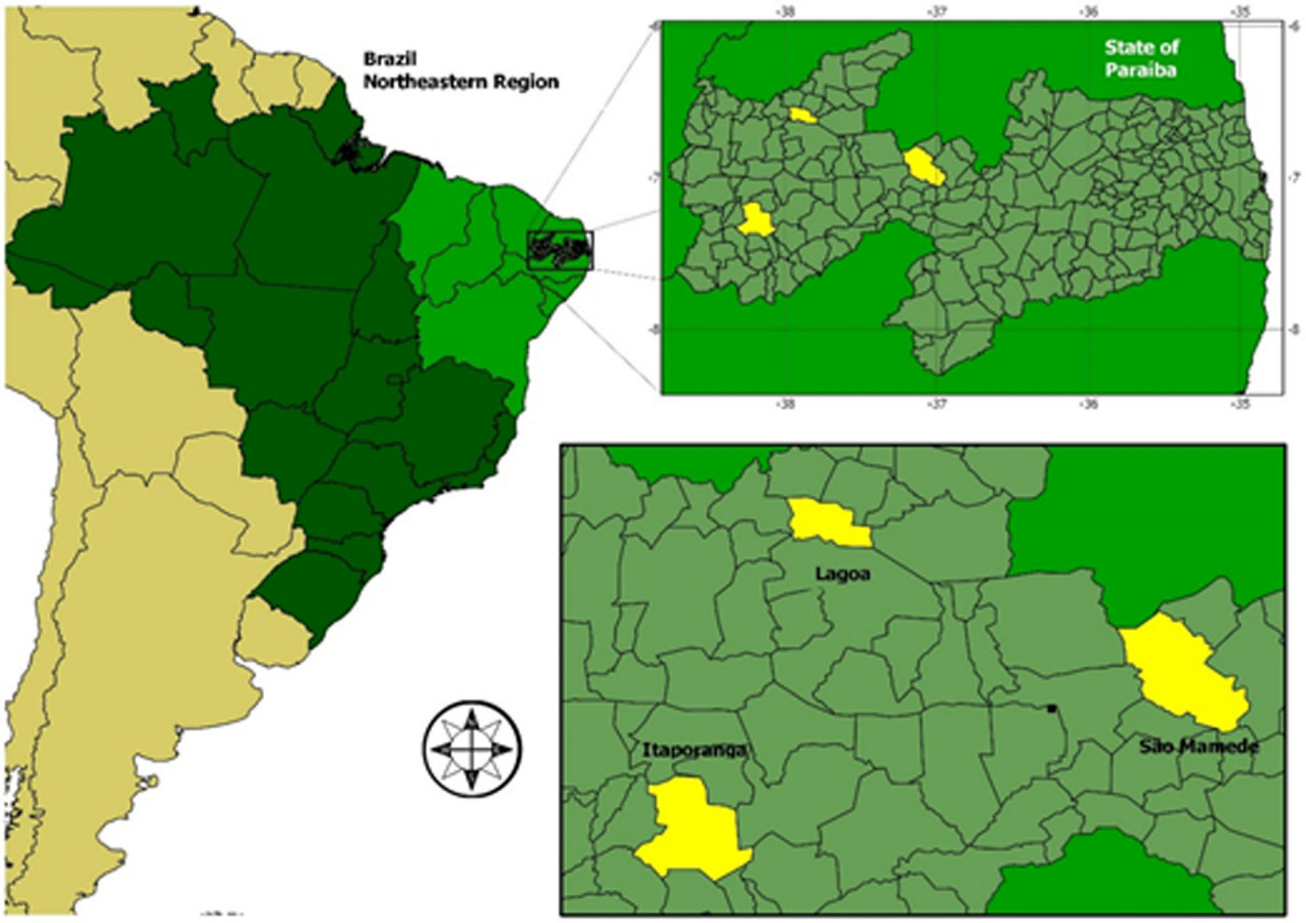
Geographic location of the study areas: São Mamede, Lagoa, and Itaporanga (Paraíba State, Northeast Brazil)

This study conducted in the rural communities of Várzea Alegre (municipality of São Mamede), Barroquinha (Lagoa), and Pau D’Arco (Itaporanga), Paraíba State, Northeast Brazil (Fig. 1). These areas were chosen because they belong to the semiarid region of Paraíba, characterized by the predominance of Caatinga vegetation, very rich in species with food potential [13–18], and geographically located in the semiarid depression, with similar edaphoclimatic influences, with average rainfall of about 350 mm per year.

The clime is BSh, characterized as a climate of hot steppes of low latitude and altitude, with an average annual temperature of 27 °C [36]. Other ethnobotanical studies had already been conducted in these communities; this fact facilitated access and the trust of residents [14, 16, 18].

### Várzea Alegre (São Mamede)

The municipality of São Mamede is at an altitude of 263 m, at the geographic coordinates 06° 55′ 37″ S and 37° 05′ 45″ W, and had an average annual rainfall of 367.5 mm in 2012 [37]. It has a population of 7548 inhabitants, covering a land area of 530,725 km^2^ [38]. São Mamede is approximately 278 km from João Pessoa (the state capital) and can be accessed by the BR-230 federal main road in the east-west direction. It borders the municipalities of Ipueira (Rio Grande do Norte State) and Várzea (Paraíba State) to the north; Várzea and Santa Luzia (Paraíba State) to the east; Areia de Baraúnas, Passagem and Quixaba to the south; and Patos and São José de Espinharas to the west (Paraíba State) [18].

The rural community of Várzea Alegre is approximately 6 km from downtown São Mamede. The local economy based on livestock, especially dairy cattle, and extensive goat and poultry breeding. In agriculture, corn and beans predominate, produced only as rainfed agriculture (in which agriculture production occurs only during the rainy season) and mostly focused on family subsistence [18]. In this community, 36 informants were interviewed, 19 women (52.78%) and 17 men (47.22%), aged 21–75 years.

### Barroquinha (Lagoa)

The municipality of Lagoa is at an approximate altitude of 281 m, at the geographic coordinates 06° 34′ 15″ S and 37° 54′ 57″ W, and had an average annual rainfall of 281.1 mm in 2012 [37]. It has a population of 4681 inhabitants and a land area of 177,901 km^2^ [38]. The municipality is approximately 394 km from João Pessoa and can be accessed by the BR-325 and BR-230 federal main road. It borders the municipalities of Bom-Sucesso, Jericó and Mato Grosso (north); Pombal (south); Paulista (east); and Santa Cruz (West).

Among the communities that comprise the rural area of the Municipality of Lagoa, Barroquinha stands out along with the communities of Jatobá, Timbaúba, and Jutubarana, due to their influence on the economy of the municipality. The community characterized by agricultural activities with small family farming areas, with corn, bean, tobacco and cotton crops, as well as sheep, goat, and cattle breeding. In this community, 66 informants were interviewed, 41 women (62.12%) and 25 men (37.88%), aged 23–79 years.

### Pau D’Arco (Itaporanga)

The municipality of Itaporanga is at an altitude of 191 m, at the geographic coordinates 07° 18′ 14″ S and 38° 09′ 00″ W, and had an average annual rainfall of 388.7 mm in 2012 [37]. It has a population of 23,192 inhabitants and a land area of 468 km^2^ [38]. The municipality is about 420 km from João Pessoa and be accessed by the BR-230 and BR-361 federal main road. It borders the municipalities of Aguiar and Igaracy (north); Boa Ventura, Diamante and Pedra Branca (south); São Jose de Caiana (east) and Piancó; and Santana dos Garrotes (west) [16].

The community of Pau D’Arco is approximately 8 km from downtown Itaporanga and consists of eight inhabited residences. Due to the lack of rain in the region, an artesian well was built, through a government action, to meet the water needs of the residents. Some residents work as day labourers on local farms and in other neighboring communities. Most of them work in the town, and some women are teachers in the municipal school [16]. In this community, 15 informants were interviewed: 7 women (46.66%) and 8 men (53.33%), aged 22–76 years.

### Data collection

The first contact with the communities was organized by health agents who work in the region; they informed all residents about the purpose and the importance of our study. The aim of this study was explained to each informant, who was then asked to sign the consent form required by the National Health Council and the Research Ethics Committee (resolution 196/96). This study was approved by Ethic Committee for Research with Human Beings of the Lauro Wanderley Hospital, in the Federal University of Paraíba, registered with CEP/HULW Protocol No.: 297/11.

All the householders from each community were interviewed, who were chosen in a directional, intentional and non-random way, seeking a balance of results between specialists and non-specialists, totaling 117 informants: 67 women (57.26%) and 50 men (42.73%). About the analysis of the food category, we considered information about plants used as food resource and its preparation forms.

Semi-structured interviews were conducted to obtain the ethnobotanical information [39]. The form addressed questions about the consumption of native food plants by humans, their uses, and which parts of these plants used. The plants cited by informants were collected, identified, classified, and processed in the field, and then they were herbarized and incorporated at the Herbarium Jaime Coelho de Moraes (EAN) of the Agricultural Sciences Center of the Federal University of Paraíba in the municipality of Areia, Paraíba State.

### Data analysis

Initially, the total richness of species listed for each community was estimated. The Jaccard index (J) was used to evaluate the similarity among the communities [40], based on the presence or absence of species in each one, and correlated the total number of species in common.

The use value index (UV) was used to determine the most important local species [41], calculated using the formula UV = Ui/*n*, in which Ui = number of use citations mentioned by each informant, and *n* = total number of informants [42]. The distinction between current and potential use citations was adopted to calculate the UV, in which the UV_current_ represents the uses that are part of the daily lives of people—these kinds of uses also called effective uses—and the UV_potential_, which represents the uses that are recognized but no longer practiced. This distinction was made during interviews asking the informants to indicate which uses were effective or not [43]. Since the samples were not normally distributed, the Kruskal-Wallis test was used to compare the UV_general_, UV_current,_ and UV_potential_ among the communities. This test was also used to compare the use values among the communities according to gender. Comparisons of use values between men and women were made using the Wilcoxon test. All analyses were performed with R [44], version i386 3.1.2, through the Rcmdr package [45].

Finally, we calculated the consensus factor among the informants (CFI), in which CFI = the number of use citations in each subcategory divided by the number of species used as food for that subcategory. These subcategories were adopted according to the uses cited by the informants, for example, cake, juice, flour, sweets, and fruit in nature. Such subcategories appear to be a way of optimizing the food potential, i.e., making them into products that have a longer shelf life, or improving the flavor and aroma of the plant. Fruits consumed directly from the plant, without any form of processing, were called consumption in nature. The consensus factor among the informants was calculated to show whether the informants are in accordance with the subcategories of use and the species cited by them.

The species citation and UV were compared among the communities using Kruskal Wallis and were compared between genders using Wilcoxon test, because the data were not normal distributed.

## Results

### Standards for food species

The results show we recorded 9 species belonging to 8 genera and 8 families in Várzea Alegre; 10 species, 9 genera, and 9 families in Barroquinha; and 7 species, 7 genera, and 7 families in Pau D’Arco (Table 1).

**Table 1:** Species of native food plants considered helpful by the inhabitants from the rural communities of Várzea Alegre, Barroquinha, and Pau D’Arco, Northeast Brazil, with their general, current, and potential use values (UV)

Community	Species	Vernacular Name	Herbarium Number	Plant Part Used	Food Use	UV_general_	UV_current_	UV_potential_
Várzea Alegre	Anacardiaceae							
	*Spondias mombin* L.	Cajarana	–	Fr	In nature, juice and sweet	0.19	0.15	0.04
	*Spondias tuberosa* Arruda	Umbuzeiro	17.556	Fr, Ro	Sweeties, juice, jam, ice, edible, umbuzada, cookies, and in nature.	1.93	1.22	0.70
	Arecaceae							
	*Copernicia prunifera* (Mill.) H.E.Moore	Carnaúba	17.553	Fr	In nature	0.15	0.04	0.11
	Capparaceae							
	*Crateva tapia* L.	Trapiá	–	Fr	In nature	0.04	–	0.04
	Euphorbiaceae							
	*Cnidoscolus quercifolius* Pohl	Favela White	17.581	Fr, Se	Flour	0.48	0.41	0.07
	Fabaceae							
	*Libidibia ferrea* (Mart. ex Tul.) L.P.Queiroz	Jucá	17.639	Fr, Se	Flour	0.04	0.04	–
	Olacaceae							
	*Ximenia americana* L.	Ameixa	17.557	Fr	In nature and juice	0.04	–	0.04
	Rhamnaceae							
	*Ziziphus joazeiro* Mart.	Juazeiro	17.575	Fr	In nature	0.38	0.19	0.19
	Sapotaceae							
	*Sideroxylon obtusifolium* (Roem. & Schult.) T.D.Penn.	Quixabeira	17.625	Fr	In nature	0.07	–	0.07
Barroquinha	Anacardiaceae							
	*Spondias mombin* L.	Cajarana	–	Fr	Juice, sweeties, and in nature	0.12	0.06	0.06
	*Spondias* sp.	Cajazeira	–	Fr	Juice and in nature	0.22	0.17	0.05
	Arecaceae							
	*Syagrus oleracea* Mart.	Côco Catolé	17.567	Fr	In nature	0.11	–	0.11
	Capparaceae							
	*Crateva tapia* L.	Trapiá	–	Fr	In nature	0.06	–	0.06
	Fabaceae							
	*Hymenoca courbaril* L.	Jatobá	17.582	Fr	In nature	0.12	0.06	0.06
	Myrtaceae							
	*Eugenia uvalha* Cambess.	Ubáia	–	Fr	In nature	0.06	–	0.06
	Olacaceae							
	*Ximenia americana* L.	Ameixa	17.557	Fr	In nature	0.12	0.06	0.06
	Passifloraceae							
	*Passiflora foetida* L.	Canapú	–	Fr	In nature	0.06	–	0.06
	Rhamnaceae							
	*Ziziphus joazeiro* Mart.	Juazeiro	17.575	Fr	In nature	0.22	0.06	0.17
	Sapotaceae							
	*Sideroxylon obtusifolium* (Roem. & Schult.) T.D.Penn	Quixabeira	17.625	Fr	In nature	0.12	0.06	0.06
Pau D’Arco	Anacardiaceae							
	*Spondias mombin* L.	Cajarana	–	Fr	In nature	0.07	0.07	–
	Arecaceae							
	*Copernicia prunifera* (Miller) H.E.Moore	Carnaúba	17.553	Fr	In nature	0.36	0.07	0.29
	Burseraceae							
	*Commiphora leptophloeos* (Mart.) J.B.Gillet	Umburana	17.642	Fr	In nature	0.21	0.07	0.14
	Capparaceae							
	*Crateva tapia* L.	Trapiá	–	Fr	In nature	0.21	–	0.21
	Fabaceae							
	*Amburana cearensis* (Allemão) A.C.Sm.	Cumarú	17.638	Fr	In nature	0.07	–	0.07
	Olacaceae							
	*Ximenia americana* L.	Ameixa	17.557	Fr	In nature	0.93	0.79	0.14
	Rhamnaceae							
	*Ziziphus joazeiro* Mart.	Juazeiro	17.575	Fr	In nature	0.43	0.29	0.14

Used parts: *Fr* fruit, *Ro* root, *Se* seed

The similarity among the communities was low. Regarding the richness of known species, Várzea Alegre and Pau D’Arco were the most similar communities (*J* = 0.455), and Pau D’Arco and Barroquinha presented the lowest similarity (*J* = 0.308). The similarity between Barroquinha and Várzea Alegre was *J* = 0.357.

In Várzea Alegre, the most prominent species were *Spondias tuberosa* Arruda, with 52 use citations; *Cnidoscolus quercifolius* Pohl, with 13 citations; and *Ziziphus joazeiro* Mart., with 10 use citations (Table 1). In this community, the fruit was the most used part and had 34 citations (38.20% of the informants) and, in nature, was the main form of consumption, with 20 citations (22.47% of the informants).

In Barroquinha, *Spondias* sp. and *Z. joazeiro* Mart. stood out, both with 4 use citations and the fruit was the plant part most used and consumed in the human diet, with 21 citations (100%) and, in nature, was the main form of consumption, with 20 citations (95.23%).

In Pau D’Arco, *X. american* L. stood out with 13 citations, followed by *Z. joazeiro* Mart. with 6 citations and *C. prunifera* (Mill.) H.E.Moore with 5 citations (Table 1). Among the species cited in this community, the fruit was the plant part most used and consumed in the human diet, with 32 citations (100% of the informants) and, in nature, was the main form of consumption (100% of the informants).

**Fig. 2:**
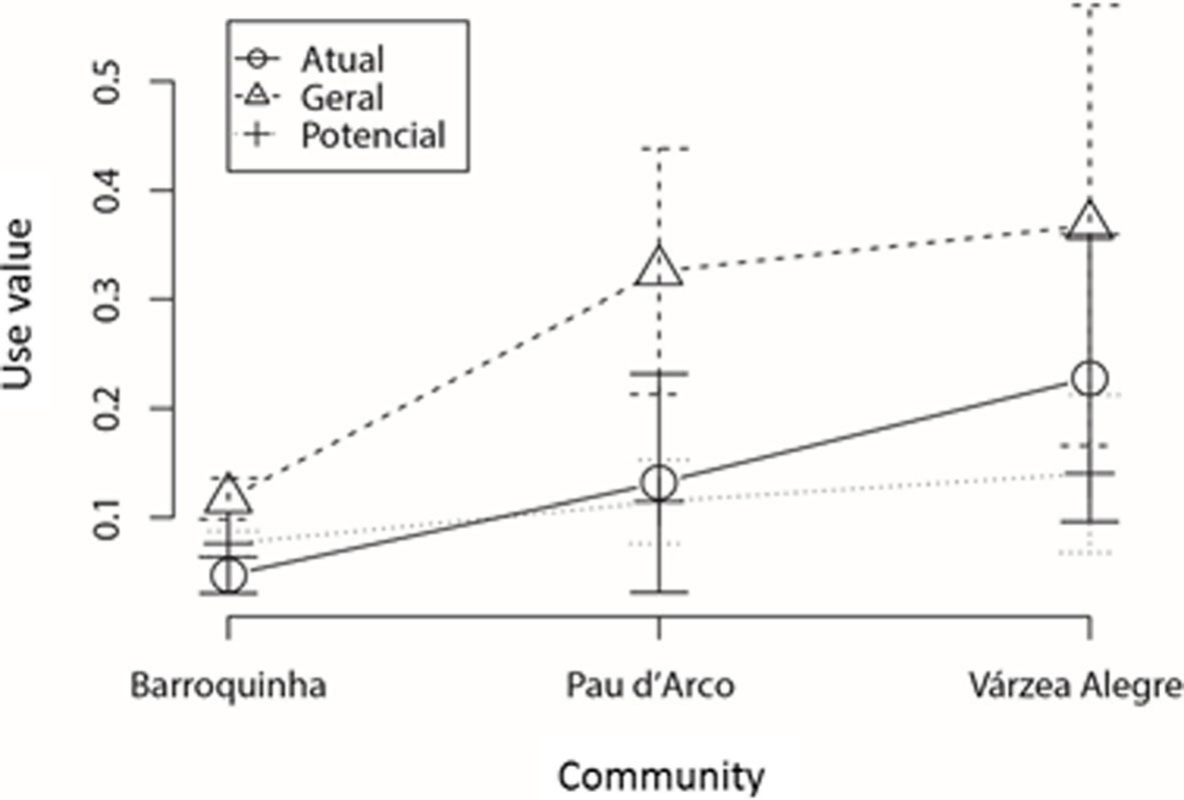
Average and standard deviation of the use value indexes (general, current, and potential) in the rural communities of Várzea Alegre, Barroquinha, and Pau D’Arco, Northeast Brazil

Considering the use values (UV_general_, UV_current_, and UV_potential_) among the three communities, there was no significant difference (*p* < 0.05) (Várzea Alegre: UV_general_ = 0.36 ± 0.60, UV_current_ = 0.22 ± 0.39, and UV_potential_ = 0.14 ± 0.21; Barroquinha: UV_general_ = 0.11 ± 0.05, UV_current_ = 0.04 ± 0.05, and UV_potential_ = 0.07 ± 0.03; Pau D’Arco: UV_general_ = 0.32 ± 0.29, UV_current_ = 0.13 ± 0.26, and UV_potential_ = 0.11 ± 0.10). There was high variability among the communities, and no difference among the medians (Fig. 2). It is important to note that the community of Barroquinha had the lowest values for the three indexes.

### Consensus factor between the informants and preparation methods

Preparation methods are slightly different in the three communities. In Várzea Alegre, the consensus factor among informants (CFI), divided into emic subcategories cited by them were, cake, coconut candy and umbuzada (1.0), sweets and flour (0.90), in nature (0.81), and juice (0.60) (Table 1). In Barroquinha only juice (CFI = 0.60) and in nature (CFI = 0.52) were cited as preparation subcategories. In Pau D’Arco, only the subcategory in nature (CFI = 0.80) was cited.

### Gender and knowledge of food plants

In the community of Várzea Alegre, both men and women attributed greater use values to *S. tuberosa* Arruda, *C. quercifolius* Pohl and *Z. joazeiro* Mart. In Barroquinha, it was recorded a predominance of *Spondias* sp*.* and *Z. joazeiro* Mart. and among women and men, *X. american* L. stood out.

In Pau D’Arco, *X. american* L. was predominant for both men and women, followed by *C. prunifera* (Mill.) H.E.Moore and *Z. joazeiro* Mart. (Table 2).

**Table 2:** Use value for men and women from the rural communities of Várzea Alegre in São Mamede, Barroquinha in Lagoa, and Pau D’Arco in Itaporanga (Northeast Brazil)

Community	Species	Vernacular Name	Women			Men		
			UV_general_	UV_current_	UV_potential_	UV_general_	UV_current_	UV_potential_
Várzea Alegre	*Ximenia americana L.*	Ameixa	–	–	–	0.08	–	0.08
	*Spondias mombin L.*	Cajarana	0.29	0.21	0.08	0.08	0.08	–
	*Copernicia prunifera* (Mill.) H.E.Moore	Carnaúba	0.14	0.07	0.07	0.15	–	0.15
	*Cnidoscolus quercifolius* Pohl	Favela	0.57	0.50	0.07	0.38	0.31	0.07
	*Ziziphus joazeiro Mart.*	Juazeiro	0.36	0.07	0.29	0.38	0.31	0.07
	*Libidibia ferrea* (Mart. ex Tul.) L.P.Queiroz	Jucá	–	–	–	0.08	0.08	–
	*Sideroxylon obtusifolium* (Roem. & Schult.) T.D.Penn.	Quixabeira	0.14	–	0.14	–	–	–
	*Crateva tapia* L.	Trapiá	–	–	–	0.08	–	0.08
	*Spondias tuberosa* Arruda	Umbuzeiro	1.71	1.00	0.71	2.15	1.46	0.69
Barroquinha	*Ximenia americana* L.	Ameixa	–	–	–	0.28	0.14	0.14
	*Spondias mombin* L.	Cajarana	0.18	0.09	0.09	–	–	–
	*Spondias* sp.	Cajazeira	0.27	0.27	–	0.14	–	0.14
	*Syagrus oleracea* Mart.	Coco catolé	0.09	–	0.09	0.14	–	0.14
	*Passiflora foetida* L.	Canapú	0.09	–	0.09	–	–	–
	*Hymenaea courbaril L.*	Jatobá	0.09	0.09	–	0.14	–	0.14
	*Ziziphus joazeiro Mart.*	Juazeiro	0.27	0.09	0.18	0.14	–	0.14
	*Sideroxylon obtusifolium* (Roem. & Schult.) T.D.Penn.	Quixabeira	0.09	0.09	–	0.14	–	0.14
	*Crateva tapia* L.	Trapiá	0.09	–	0.09	–	–	–
	*Eugenia uvalha* Cambess.	Ubáia	–	–	–	0.14	–	0.14
Pau D’Arco	*Ximenia americana* L.	Ameixa	1.00	1.00	–	0.88	0.63	0.25
	*Spondias mombin* L.	Cajarana	–	–	–	0.13	0.13	–
	*Copernicia prunifera* (Mill.) H.E.Moore	Carnaúba	0.50	0.17	0.33	0.25	–	0.25
	*Amburana cearensis* (Allemão) A.C.Sm.	Cumarú	0.17	–	0.17	–	–	–
	*Ziziphus joazeiro Mart.*	Juazeiro	0.50	0.33	0.17	0.38	0.25	0.13
	*Crateva tapia* L.	Trapiá	0.17	–	0.17	0.25	–	0.25
	*Commiphora leptophloeos* (Mart.) J.B.Gillet	Umburana	0.34	0.17	0.17	0.13	–	0.13

About the distribution of knowledge, taking into consideration the forms of use and the number of citations, there were no differences regarding the knowledge that men and women have about food plants within each community (Table 3). Considering the same gender among the communities, man and women in Várzea Alegre post have more knowledge about the plants than man and women in the other two communities (Pau D’Arco and Barroquinha) (Table 3).

**Table 3:** Distribution of traditional knowledge by gender among rural communities in the semiarid region of Paraiba State, Brazil

Community	Genre	Methods of use Χ ± SD	Citations Χ ± SD
Várzea Alegre	Women	1.85 ± 0.94^aA^	3.21 ± 1.96^aA^
	Men	2.30 ± 2.17^aA^	3.38 ± 2.72^aA^
Pau d’Arco	Women	1.00 ± 0.00^aB^	2.66 ± 1.03^aAB^
	Men	1.00 ± 0.00^aB^	2.00 ± 1.41^aAB^
Barroquinha	Women	1.09 ± 0.30^aB^	1.18 ± 0.40^aB^
	Men	1.00 ± 0.00^aB^	1.14 ± 0.37^aB^

Equal lowercase letters in the same column, within each region between genders, indicated the absence of significant differences, using the Mann-Whitney test (*p* < 0.05)

Different capital letters in the same column among regions for the same gender indicate significant differences, using the Kruskal-Wallis test (*p* < 0.05)

**Fig. 3:**
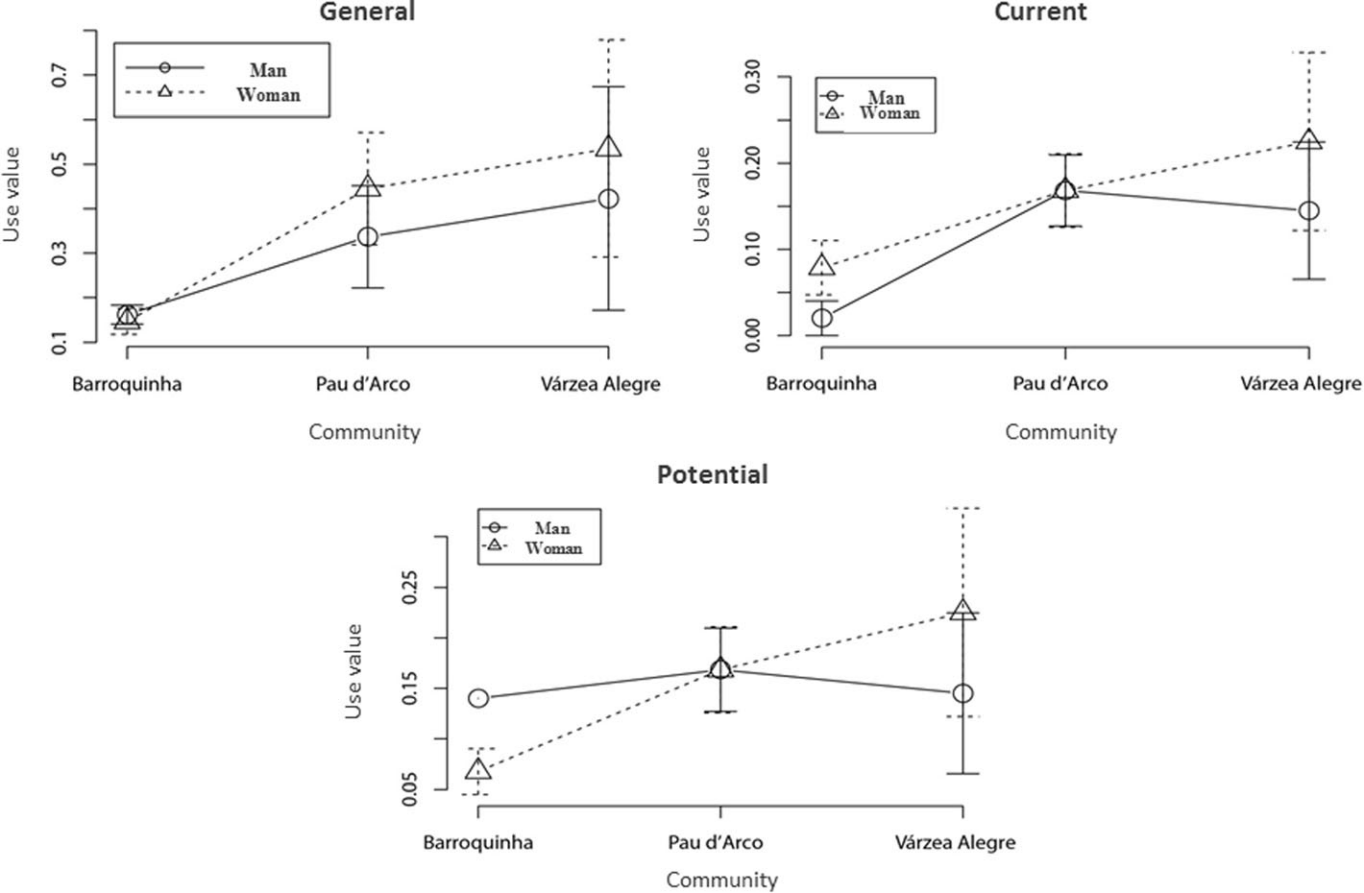
General, current, and potential use values, considering gender, in the rural communities of Várzea Alegre, Barroquinha, and Pau D’Arco, Northeast Brazil

Men and women have higher knowledge of species used as food in Várzea Alegre than in Barroquinha. In general, there was no difference between Pau D’Arco and any other community (Table 3). The use value analysis according to gender among the communities showed significant differences only for UV_general_ among women (*p* < 0.05), specifically between Barroquinha and Pau D’Arco (H = 7.89, *p* < 0.05) (Fig. 3).

Regarding the use value index between men and women within each community, there were differences just for UV_potential_ in Barroquinha (*W* = 49, *p* < 0.05) (Fig. 3).

## Discussion

Many studies about the use and knowledge of plant species in communities located in the semiarid region of Paraíba State identified species with potential for use in the human diet [13–18]. However, in the communities studied here, we found limited knowledge regarding the food potential of native Caatinga plants.

This fact evidenced by the low richness of species cited by the informants, with an average of 8.66 species per community. We can evidence this limited knowledge by comparing our study to another conducted in the municipality of Soledad, also in Paraíba State, in which, in only one community, more than 30 native species with use and food potential were cited [13]. The difference regarding knowledge among the communities may be associated with factors such as the presence of exotic species, proximity to urban areas, which facilitates access to other types of food, or because of the low diversity of species with food potential due to the low rainfall.

Several studies performed on a global scale, also aimed at recording the knowledge of food plants, showed a high number of species, such as the studies carried out in Xi’iuyem communities in San Luis Potosi, Mexico (BSk climate, cold steppes of mid-latitude and high altitude), where more than 50 species were identified, especially those from the families Fabaceae, Solanaceae, and Cactaceae, a very higher number in comparison with our study [46].

An ethnobotanical study conducted in flooded areas of rivers in northern China, recorded over 100 species and subspecies of food plants, especially from the families Rosaceae, Zingiberaceae and Solanaceae [47], showing a high diversity of species, possibly due to the proximity to the water, and soil fertility.

In accordance with previous studies [13, 18], *S. tuberosa* Arruda stood out in the community of Várzea Alegre as one of the major native food plants of Caatinga, with many citations, but it was not mentioned in Barroquinha and Pau D’Arco. In Paraíba, *S. tuberosa* Arruda predominates in the Borborema Plateau region and around it, but rarely found in the depression of the semiarid region. The fruits of this species are consumed in nature and used to make juice, sweets, cake, and umbuzada.

*C. quercifolius* Pohl was another food potential species cited in Várzea Alegre; this species found in vegetation more far from the residences, in more densely populated forests. Its pods release seeds that have low moisture and not a pleasant taste, but its use in the human diet has already been reported, especially the use of its ground seeds as flour [48], which increases its availability for longer, ensuring the presence of this food in times of dire need, due to its low moisture content.

The biggest difference reported between *S. tuberosa* Arruda and *C. quercifolius* Pohl in the community of Várzea Alegre is possibly the higher availability of *S. tuberosa* Arruda fruit, especially near the residences and on the roads, and its versatility of consumption in addition to the good taste and aroma of its pulp, in comparison to *C. quercifolius* Pohl. Additionally, the consumption of *S. tuberosa* Arruda fruits has high importance for the regions where it occurs because it has a significant content of ascorbic acid, yellow flavonoids, and carotenoids; these compounds give this species considerable antioxidant activity and recognized functional properties, which in the future could be exploited in both the food industry and the pharmaceutical industry [49, 50].

Studies conducted in the semiarid region of Pernambuco [13, 51] evidenced that food plants near to the residences, or easy to access, with good taste and aroma, such as *Spondias* sp. and *Z. joazeiro* Mart., are preferred by the residents, and this fact explains the number of citations of these species by the informants.

Due to the economic importance of *S. tuberosa*, some studies conducted aiming at its fruit cultivation, development, harvesting, and processing. In spite of these studies on the economic exploitation of *S. tuberosa* Arruda, there is still a lack of studies on the floral biology and pollination of this species [52–55]. In the semiarid region of Northeast Brazil, it evidenced that humans are conducting an incipient management of this species, changing the structure of ecosystems where it grows, and even performing an artificial selection of genetic material [56]. These traditional processes, locally developed, can be extremely significant for possible domestication processes and consequently production on commercial scales. These studies are extremely important for the conservation of the species, given that their exploitation occurs in an extractive way, in many regions where they occur naturally, without determining the pollinators of the species and the level of dependence between them and the formation of fruit [57].

In Barroquinha, only the community in where *Spondias* sp. and *Z. joazeiro* Mart. were mentioned together, there was equivalence for UV_general_. Thus, the UV_general_ should not be used as representing the whole, because it can dissemble some information by overvaluing or decreasing the importance of the used species. Hence, the importance of visualizing such indexes is separated into UV_current_ and UV_potential_ [43].

Analyzing UV_current_ for all cited species, it can be noticed that the informants more effectively use *Spondias* sp. than *Z. joazeiro* Mart., although this study reveals that the informants know the food potential of these species. *Z. joazeiro* Mart. is prominent in the human diet as it presents a good relationship between soluble solids and acidity, which is directly associated with a pleasant taste. This species seems to be a high importance for studies on the agro-industrial potential, and it can be an alternative to complement the income of the local population, which confirmed in studies in the semiarid region of Paraíba [13].

The fruit of *Spondias* sp. has carotenoid pigments precursors of vitamin A, whose content is higher than that found in mango (*Mangifera indica* L*.*), and it is recognized by its high antioxidant activity [49]. In turn, the fruit of *Spondias* sp. has higher carotenoid content than the fruit of *Spondias monbin* L., which gives it a high functional potential [50] and may be valued regionally for the enrichment of school meals.

Concerning the use value among the three communities, there was no significant difference regarding the knowledge of food plants; however, in the comparison between genders among the three communities, there was a significant difference, which can be verified in other studies [58, 59]. This can be explained by the fact that men walk more effectively in the woods to hunt, fish, collect, or take care of their flocks, and women stay more around the residences, taking care of them, or going out to collect firewood and food that is the basis of the daily diet, but both men and women have knowledge same for some species and different for others [60, 61].

The hypotheses that guide this evaluation are (1) the low availability of these resources; (2) the size of the communities, making the transmission of knowledge easier; and (3) change in habits for both genders, which may be causing a loss of knowledge, motivated mainly by the ease of access to social programs promoted by the Federal Government of Brazil. Despite generating a lot of discussion and other studies, these results are similar to those found in other studies also conducted in the semiarid region of Paraíba State [13–15, 43].

It has been observed that on some occasions, people more appreciate the plants that present several preparation methods, availability, and easy acquisition than those that are more difficult to collect, due to their spines and height or even because they are too far from the residences [62], as well as the difficulty of processing them into some derivatives; the objective is eating, expending the least amount of energy as possible, even if the obtained food is not so nutritional.

All informants in Barroquinha stated the fruit is the most used plant part. Among women, *Spondias* sp*.* effectively predominated, especially as juice and sweets, and because this species is next to the residences (usually in gardens) and on the roads. Among men, knowledge of the potential of *Z. joazeiro* Mart. predominated in Barroquinha. In this community, men know and use *X. americana* L*.* as food when it is available in the vegetation, especially when they are in the woods carrying out their daily activities since this species occurs far from the residences. This fact grabs our attention on the cultural habit of men of using native species that are more distant from the residences, whereas women use the species that are nearer the residences [60–62].

In the community of Pau D’Arco, *X. americana L.* stood out for both genders. This specie is important due to its high UV_current_, which shows that the informants consume this species, especially its fruit in nature. This result was also observed for *Z. joazeiro* Mart., which had constant citations regarding the consumption of fruit in nature in this community. *C. prunifera* (Mill.) H.E.Moore and *C. tapia* L. had high UV_potential,_ confirming the potential importance that many species have as food sources, whether for an emergency or not, such as some species cited in studies conducted in Nepal [63]. Thus, it can be noticed that further studies are still needed in several areas to know and record all the potential of food plants in the semiarid region of Brazil.

## Conclusions

This study showed that the residents of the three rural communities have small knowledge of native food plants found in their communities in comparison with other studies, but they know where to find them, which parts they can use, and how to consume them. This reduced knowledge must be analyzed from different points of view: the low richness of plants with food potential in the region; the seasonality; the use of these species for other purposes such as timber, medicines, fodder, among others; and the ease of acquiring exotic species, besides the cultural inertia issues.

The knowledge of men and women is another aspect to address since in some cases species have higher importance current or potential exclusively for a gender, despite being cited by the opposite gender. The fact that men quoted plants that are more distant from the residences and women quote those that are next to them still needs to be better explained, requiring other studies with this focus. The pattern observed in this study in the three communities considering gender just reveals how complex and how unique the food selection process can be in each location. These aspects must contemplate to future programs to promote an adequate feeding.

The residents’ practical knowledge should be employed along with the scientific knowledge to develop strategies to manage these species in the best possible way, because in the near future these species may be important in the human diet, contributing to species diversification, and becoming important food and compound sources with functional properties well-known to protect our body against degenerative diseases and metabolic syndromes.

It was important to notice that we need to know more about these native species, their production, seasonality, storage methods, and pests. In addition, this study, along with other conducted in nearby regions, can serve as support for major studies aimed at the local knowledge, which should be encouraged and exploited rationally.

## Abbreviations

CFI: Consensus factor among the informants
EAN: Herbarium Jaime Coelho de Moraes
JI: Jaccard index
UV: Use value index
